# Determination of minimum inhibitory concentrations using machine-learning-assisted agar dilution

**DOI:** 10.1128/spectrum.04209-23

**Published:** 2024-03-22

**Authors:** Alessandro Gerada, Nicholas Harper, Alex Howard, Nada Reza, William Hope

**Affiliations:** 1 Antimicrobial Pharmacodynamics and Therapeutics Group, Department of Pharmacology and Therapeutics, Institute of Systems, Molecular & Integrative Biology, University of Liverpool, Liverpool, United Kingdom; 2 Department of Infection and Immunity, Liverpool Clinical Laboratories, Clinical Support Services Building (CSSB), Liverpool University Hospitals NHS Foundation Trust—Royal Liverpool Site, Liverpool, United Kingdom; Quest Diagnostics Nichols Institute, Chantilly, Virginia, USA

**Keywords:** antimicrobial resistance, minimum inhibitory concentration, artificial intelligence, machine learning, assay validation, image recognition, laboratory software, digital health

## Abstract

Effective policy to address the global threat of antimicrobial resistance requires robust antimicrobial susceptibility data. Traditional methods for measuring minimum inhibitory concentration (MIC) are resource intensive, subject to human error, and require considerable infrastructure. AIgarMIC streamlines and standardizes MIC measurement and is especially valuable for large-scale surveillance activities. MICs were measured using agar dilution for *n* = 10 antibiotics against clinical Enterobacterales isolates (*n* = 1,086) obtained from a large tertiary hospital microbiology laboratory. *Escherichia coli* (*n* = 827, 76%) was the most common organism. Photographs of agar plates were divided into smaller images covering one inoculation site. A labeled data set of colony images was created and used to train a convolutional neural network to classify images based on whether a bacterial colony was present (first-step model). If growth was present, a second-step model determined whether colony morphology suggested antimicrobial growth inhibition. The ability of the AI to determine MIC was then compared with standard visual determination. The first-step model classified bacterial growth as present/absent with 94.3% accuracy. The second-step model classified colonies as “inhibited” or “good growth” with 88.6% accuracy. For the determination of MIC, the rate of essential agreement was 98.9% (644/651), with a bias of −7.8%, compared with manual annotation. AIgarMIC uses artificial intelligence to automate endpoint assessments for agar dilution and potentially increases throughput without bespoke equipment. AIgarMIC reduces laboratory barriers to generating high-quality MIC data that can be used for large-scale surveillance programs.

**IMPORTANCE:**

This research uses modern artificial intelligence and machine-learning approaches to standardize and automate the interpretation of agar dilution minimum inhibitory concentration testing. Artificial intelligence is currently of significant topical interest to researchers and clinicians. In our manuscript, we demonstrate a use-case in the microbiology laboratory and present validation data for the model’s performance against manual interpretation.

## INTRODUCTION

Antimicrobial resistance (AMR) constrains the effective treatment of bacterial infections and is a persistent obstacle to improvements in global health (1). *In vitro* detection of AMR is a key technique in research and clinical laboratories and underpins drug development, epidemiology, and direct patient care. Strategically important global initiatives such as AMR surveillance and antimicrobial stewardship rely on antimicrobial susceptibility testing using Clinical Laboratory Sciences Institute and the European Committee on Antimicrobial Susceptibility Testing (EUCAST) methodology (2, 3).

Substantial financial and human resources are required to perform and interpret MICs, even with hardware to assist with these tasks (4). Most laboratories can only perform microdilution assays on a subset of strains. At the heart of the problem is the number of data points that must be generated and interpreted. For example, a surveillance program using agar dilution of microdilution techniques with 10 antibiotics tested at 10 dilutions on 1,000 bacterial strains generates 100,000 (1 × 10^5^) data points that need to be recorded and tabulated—a volume of workload that increases the probability of measurement errors. For example, between 2016 and 2020, the Antimicrobial Testing Leadership and Surveillance Database (ATLAS) recorded on average ~500,000 MICs annually (5 × 10^5^ data points) (5).

Recent years have seen the increasing application of artificial intelligence (AI) to workflows within clinical microbiology laboratories. Applications include the interpretation of microscopy images (e.g., Gram stain) and bacterial colonial morphology (6). Image recognition algorithms are now routinely integrated into some clinical laboratory automation systems (7). As AI becomes increasingly available and accessible, it is expected to have a disruptive effect on laboratory diagnostics (8). There is also increasing interest in the role of AI in assisting clinical microbiology laboratories that do not have access to automated platforms for antimicrobial susceptibility testing. For example, the Médecins Sans Frontières-funded Antibiogo smartphone application is used in clinical microbiology laboratories for disk susceptibility testing interpretation using image recognition (9).

Here, we describe AIgarMIC, a software package that combines convolutional neural network image recognition with expert rules to enable moderate throughput semi-automated measurement of MIC at low cost. We outline the training, validation, and proposed operation of AIgarMIC using a modified agar dilution protocol and serial plate imaging to produce a colony growth matrix that is used to calculate MICs.

## MATERIALS AND METHODS

### Organisms used in this study

Gram-negative Enterobacterales bacterial strains (*n* = 1,086), predominantly *Escherichia coli* (*n* = 827, 76%), were retrieved from long-term −80°C storage (Table 1). To ensure a wide range of MICs and likely resistance mechanisms were captured, most of the strains (*n* = 745, 69%) were selected by cluster sampling of stored isolates (from any clinical site) from a large clinical laboratory servicing the city of Liverpool, UK (Liverpool Clinical Laboratories, UK). First, we collected a list of all stored *E. coli* isolated from any clinical site between 2017 and 2021. Next, strains were grouped into eight broad phenotypes (based on the disk susceptibility reported by the laboratory using EUCAST methodology, v.7.1–v.11.0), and samples without replacement were generated equally from each group. The rest of the isolates (*n* = 341, 31%) were from unrelated research programs with a heterogeneous mix of species. All isolates were identified using MALDI-TOF (Bruker Daltonics, Massachusetts, USA).

**TABLE 1:** Strains used in this study

Species	*n*	%
*Escherichia coli*	827	76%
*Klebsiella* spp.	98	9%
*Enterobacter* spp.	105	10%
*Klebsiella pneumoniae*	31	3%
*Klebsiella aerogenes*	5	0%
*Citrobacter* spp.	9	1%
Other gram-negative	11	1%
Total	1,086	

### Overall strategy and workflow

**Fig 1:**
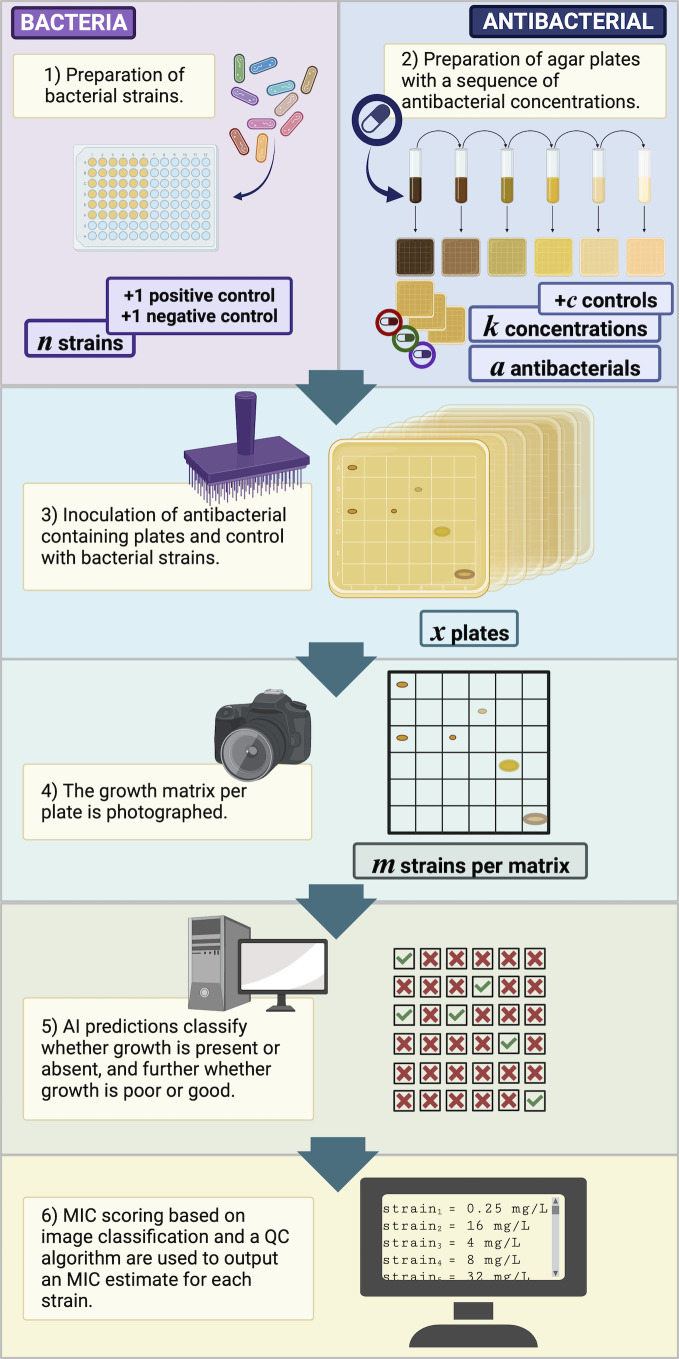
A summary of the steps required to develop AIgarMIC, where *c* is the number of control (negative) plates, *k* is the number of concentrations per antimicrobial, *a* is the number of antimicrobials, *x* is the total number of plates required, and *m* is the number of strains/spots per plate (up to 96 in our study).

The overall strategy to develop and validate an AI for the determination of MICs using agar dilution is shown in Fig. 1.

### Agar dilution methodology

#### Preparation of plates

To determine MICs, we adapted an agar dilution protocol described elsewhere (10).

Isolates were tested against amikacin, chloramphenicol, ciprofloxacin, gentamicin, meropenem, amoxicillin, amoxicillin/clavulanic acid, cefepime, ceftazidime, flomoxef, and tigecycline (MERCK, Poole UK) using a series of doubling dilutions of each drug in Mueller-Hinton agar (Fisher Scientific, Loughborough UK). Antimicrobial-containing agar was poured into Corning 120 cm × 120 cm square plates (Fisher Scientific), allowed to cool and stored at 4°C until use. Plates were used within 2 days of preparation.

#### Preparation of inoculum

Glycerol stocks of isolates were arrayed for storage in 96-well PCR plates. Each plate contained 94 isolates with the last two wells being reserved for a negative control (broth alone) and a positive control *E. coli* strain (ATCC 25922) with known MICs (11).

Plates were retrieved from −80°C storage and used to inoculate flat-bottomed 96-well plates containing 100 µL of Mueller-Hinton broth (MHB). Plates were incubated at 37°C with shaking until the cultures had reached an OD_625_ over 0.08 (within 3 h). Cultures were then adjusted to 0.5 McFarland standard turbidity (OD_625_ = 0.08) by dilution in MHB. Cultures below 0.08 were discarded.

Adjusted cultures were inoculated onto the 120 cm × 120 cm agar plates using a 96-pin manual replicator (Boekel Scientific, Philadelphia, USA) which delivers 1 µL of suspension to achieve approximately 10^4^ colony-forming units per spot. Plates were then incubated for 18 h at 37°C.

#### Determination of MIC

Growth was determined after 18 h of incubation. The MIC was taken as the plate with no discernable growth using the naked eye. Plates with isolated colonies or a faint film of growth were counted as no growth.

### Development of AI

#### Colony image labeling and classification

Photographs of plates were taken using a consumer-grade digital camera (Canon EOS 4000D). A total of 1,156 images of agar plates (each consisting of up to 94 strains growing in a fixed drug concentration) were generated by photographing agar dilution growth. Images were subsequently processed by splitting each plate image into 96 sub-images of 9 × 9 mm squares surrounding each spot.

These sub-images were used to create a labeled data set suitable for training an image classification algorithm. A clinical microbiologist (AG) labeled sub-images, assigning them to one of three classes based on the phenotypic appearance of colonies: (i) images with no growth (Class I), (ii) images of poor or inhibited growth (Class II), and (iii) images of uninhibited growth (Class III)—see Fig. 3 for examples. The inclusion of Class II (poor/inhibited growth) was necessary to accurately calculate MICs since colonies demonstrating antimicrobial inhibition (single colonies or a faint film) need to be disregarded (12). Image labeling took place prior to and separately from model construction. Once images were annotated, 20% of each of the three classes (no growth, poor/inhibited growth, and good growth) was reserved to test the performance of the classification models in identifying images.

#### Neural network architecture and machine learning

Since colony size and morphological appearance are important factors in determining whether there is healthy bacterial growth at a particular plate position, a convolutional neural network was designed to classify sub-images as no growth, poor/inhibited growth, or good growth using the labeled image data set. Models were implemented using the keras library in the Python programming language (13, 14). The OpenCV library was used for image manipulation and standardization prior to model training and prediction (15).

A multiclass neural network was initially designed, and images were assigned to one of the three classes. However, given that the difficulty of differentiating between the classes was not expected to be equally split (i.e., differentiating presence from the absence of growth is an easier problem than differentiating partially inhibited vs uninhibited growth), a two-step binary classification model was preferred. Here, an initial binary model classified images based on whether growth was present. A second-step binary model then classified images where growth was present into good growth or inhibited growth classes.

Neural network models can overfit the training data, meaning the model performs well on the training images but gives poor results on new images. To minimize this, the neural network was regularized through a combination of image augmentation (random image rotation and flipping) and early cessation of training (based on visual inspection of fitting plots) (16). Model regularization prevents overtraining and maintains generalizability of the model when applied to new data. Since bacterial colony size is an important feature of growth quality, image augmentation layers such as cropping or zooming were not used. The model was trained using the Adam optimizer and a binary cross-entropy loss function (17, 18).

To predict MICs, the neural networks were used to convert a series of agar plate images (each containing up to 96 strains plated on agar containing a fixed drug concentration) to a series of bacterial growth matrices. An algorithm was implemented that analyzed the three-dimensional matrix to identify the highest concentration with poor/inhibited or absent bacterial growth, corresponding to the MIC for each strain. Poor/inhibited growth was counted as no growth—in line with EUCAST agar dilution methodology (12).

#### Software development

A Python package suite was developed with the following key features (13):

- Automated partitioning of images into component sub-images
- Interactive manual annotation for labeled data set generation
- Neural network model training
- Calculation of MIC from a set of agar images along a concentration gradient
- Quality assurance (e.g., alerts for the absence of growth in antibiotic-free medium)
- Optional user manual prompt for images with low prediction certainty

#### Validation of colony classification algorithms

The performance of the neural networks in classifying colony images was initially tested using the 20% subset of labeled colony images. The following binary accuracy metric was used for model evaluation: $\text{Accuracy}=\frac{TP+TN}{FP+FN+TP+TN}$, where TP = true positives, TN = true negatives, FP = false positives, and FN = false negatives.

#### Validation of MIC predictions

To assess the performance of AIgarMIC in predicting MIC, 130 of the agar plate images (94 *E. coli* strains tested against 10 antimicrobials—amikacin, chloramphenicol, ciprofloxacin, gentamicin, meropenem, amoxicillin, amoxicillin/clavulanic acid, cefepime, ceftazidime, and tigecycline) were kept aside. Once the model was trained and software developed, the images were processed to predict MICs. Flomoxef was not included in this step as target MIC for *E. coli* 25922 is not available in EUCAST guidance (11). Results were compared against manual annotation by the same microbiologist that labeled the colony images (AG). The following metrics were used to compare the model’s MIC predictions to manually annotated MICs: essential agreement (MIC ± 1 dilution) and assay bias as described in ISO 20776-2:2021, minor error (intermediate on one method, susceptible/resistant on the other), major error (resistant on AIgarMIC, susceptible on manual annotation), very major error (susceptible on AIgarMIC, resistant on manual annotation) (19, 20) (11). Breakpoints were used for determining error and categorical agreement (3). The images used for the final validation process were not used to generate colony images to train the neural network. The option to manually correct sub-images with poor annotation accuracy was not used when predicting MICs using AIgarMIC.

## RESULTS

### Overall workflow

**Fig 2:**
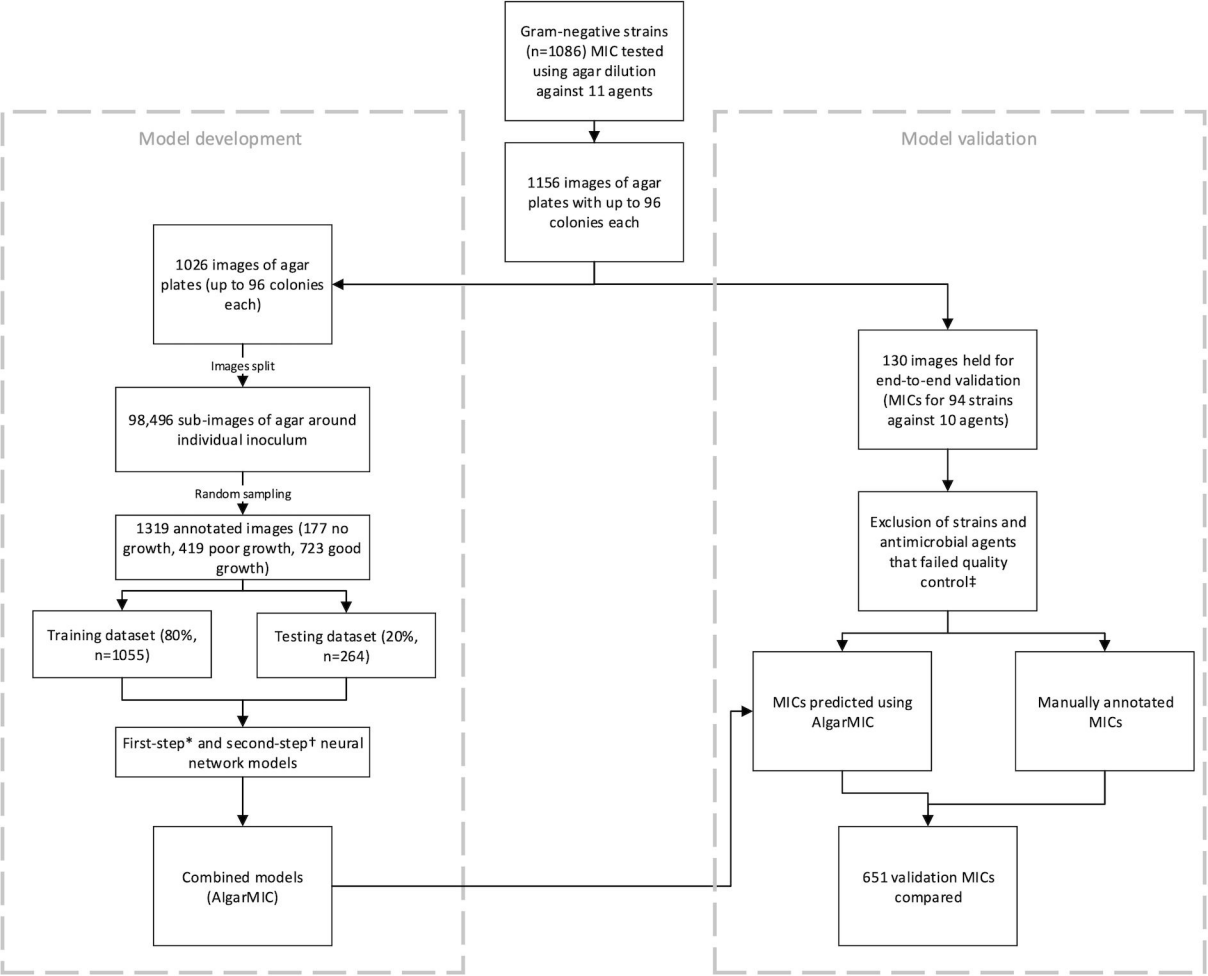
Overview of study methodology and design. *Strains that failed to grow, or agar plates in which the control strain *E. coli* 25922 MIC was outside the expected range were excluded, †no growth vs growth, ‡poor/inhibited growth vs good growth.

A summary of the workflow is shown in Fig. 2, which highlights the number of datapoints to (i) develop the algorithm and (ii) compare MICs as determined by the AI vs standard methodology.

### Development of the AI

#### Colony image classification

**Fig 3:**
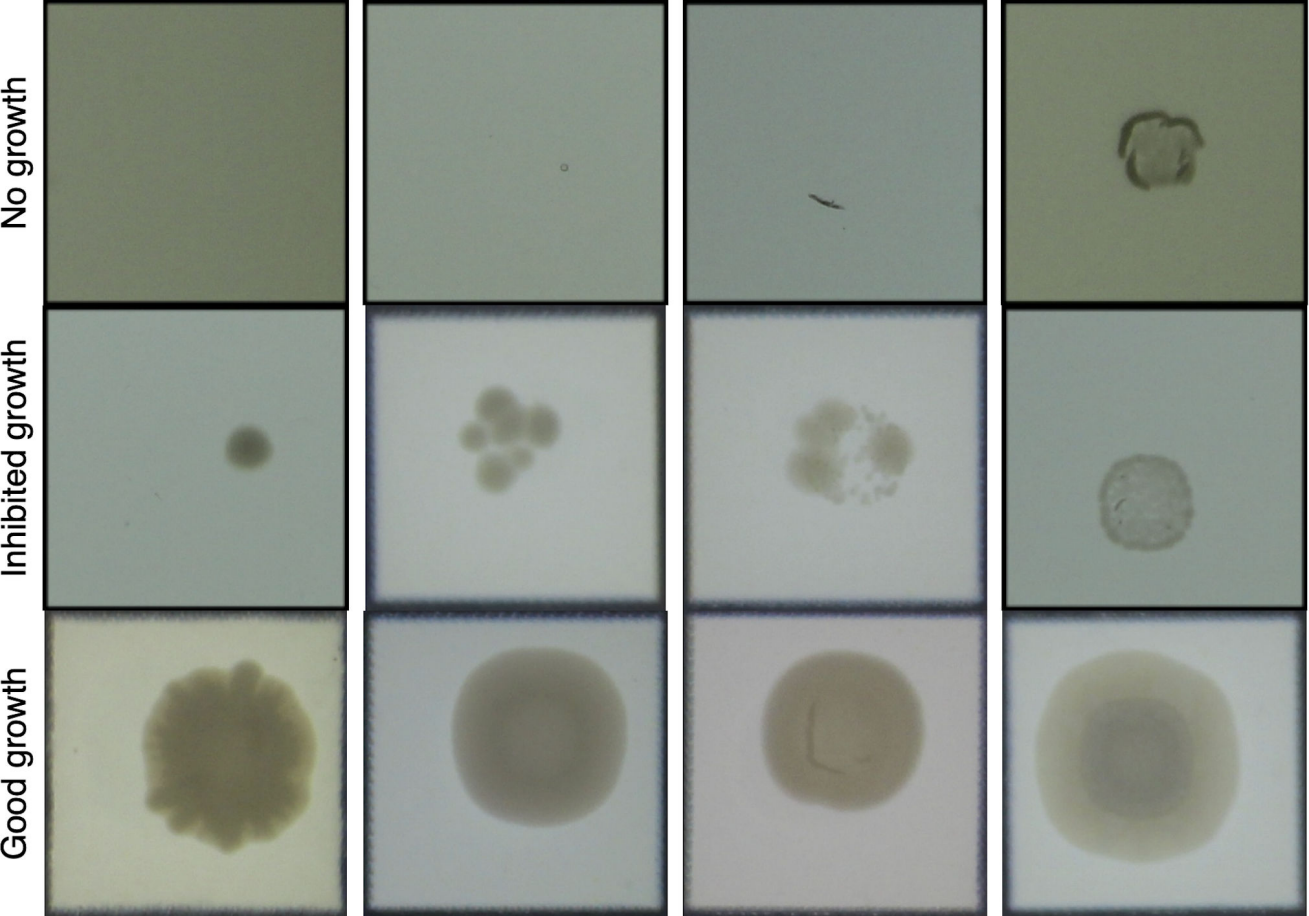
Heterogeneity of colony phenotypic morphology. No growth (top row) can include agar and imaging artifacts. Antimicrobial-inhibited growth (middle row) includes single colony (left), discrete inhibited colonies (middle), and faint film of growth (right). Growth of colonies in the bottom row is not inhibited by the tested antimicrobial.

**Fig 4:**
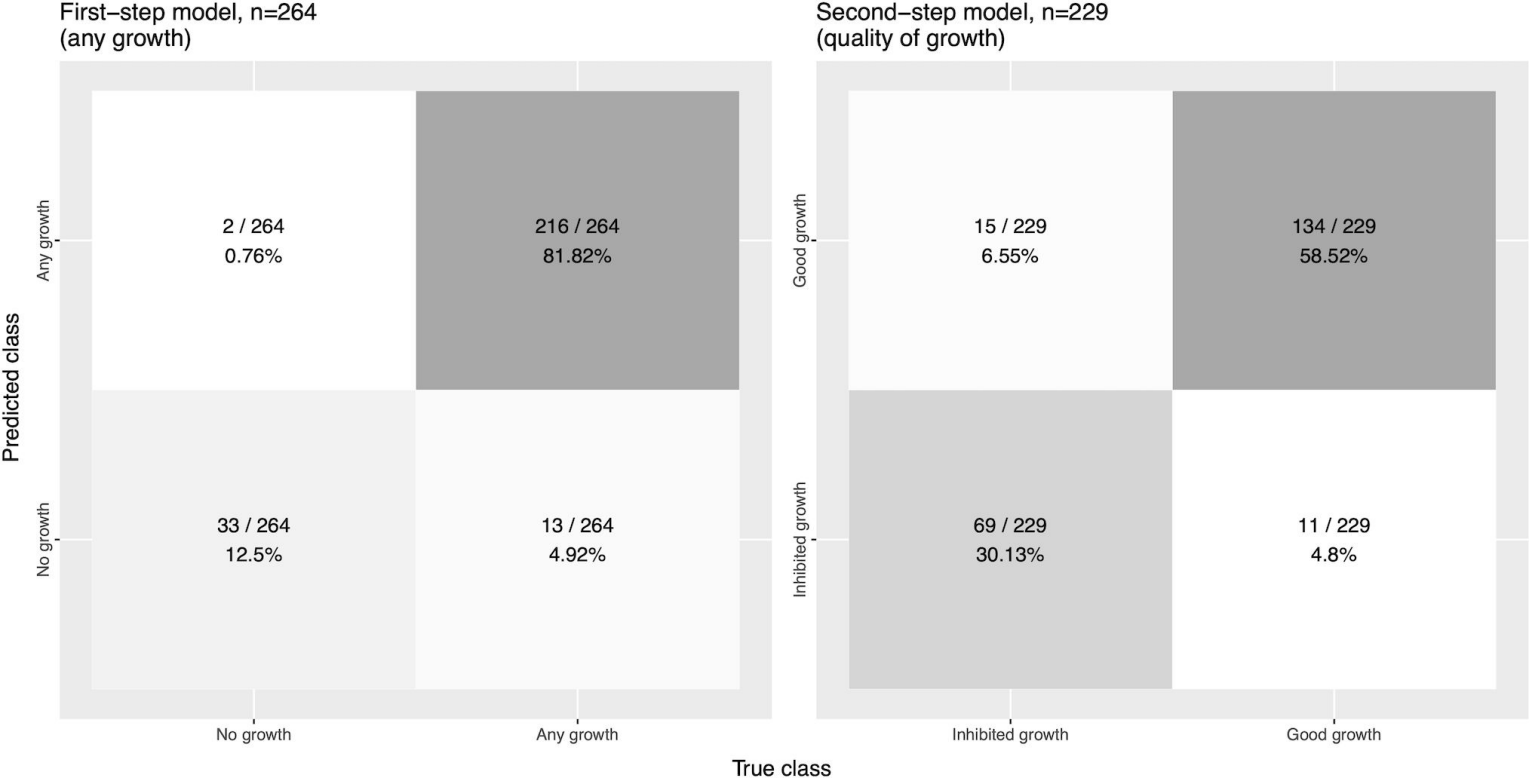
Confusion matrix for the performance of both models on the validation colony image dataset. The first-step model determines whether any bacterial growth is present in the image. The second-step model determines the quality of the growth.

The determination of growth vs no growth with the neural network model was straightforward and was achieved with 94.3% accuracy when assessed using the reserved datapoints (“testing data set”). Determination of the second-step (i.e., uninhibited vs inhibited growth) of the model was more complicated because the heterogeneity in the appearance of drug-affected colonies vs artefact (Fig. 3) required greater computational requirements to enable this distinction. Nevertheless, an accuracy of 88.6% was achieved. The performance of the AI in terms of identifying any growth and whether there was inhibited growth is summarized in Fig. 4.

#### Validation of model’s MIC predictions against manual annotation

**Fig 5:**
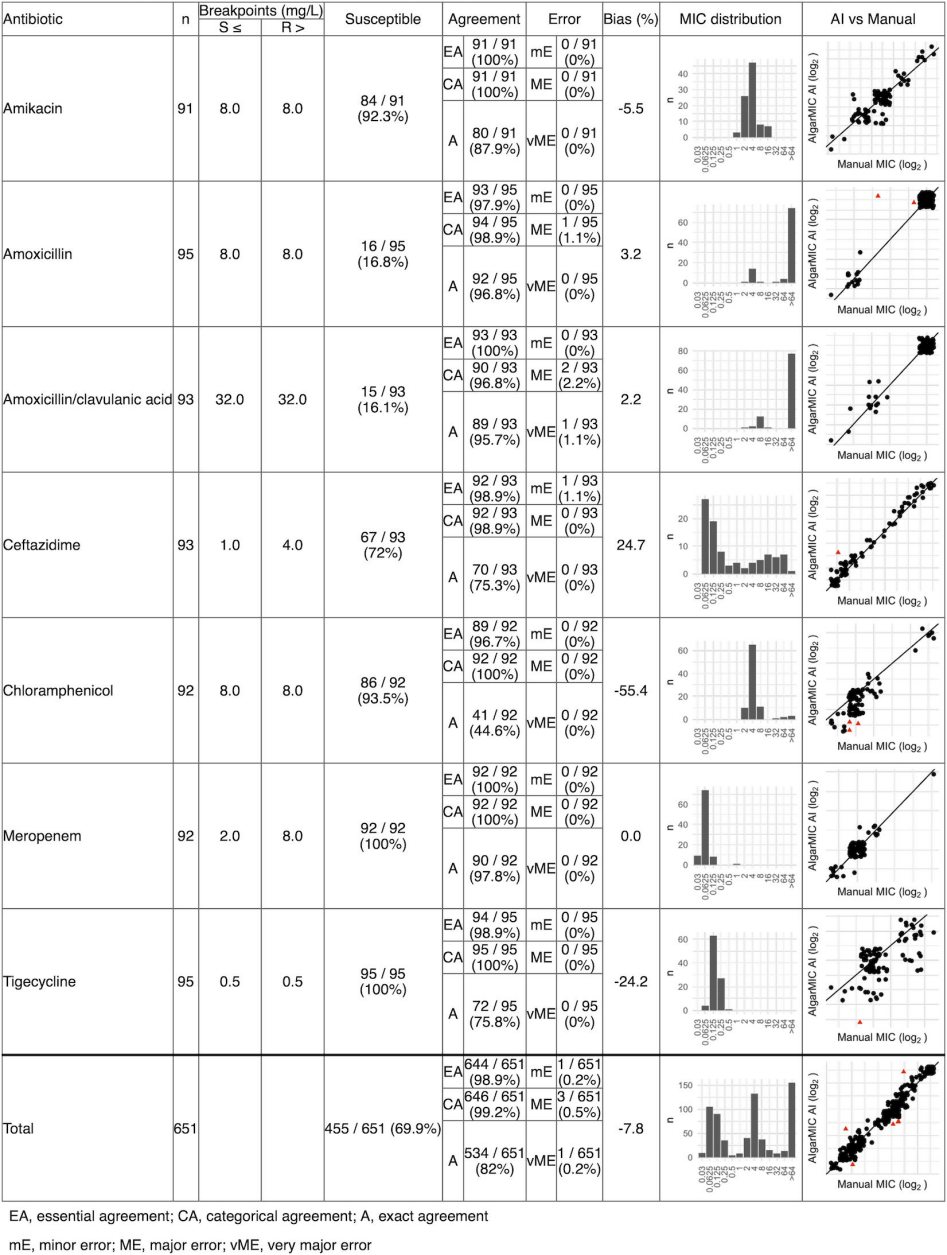
Validation summary for AIgarMIC against manual annotation on *E. coli* strains. Red triangles in right-hand plot indicate MICs that failed essential agreement. The diagonal line is the line of identity (i.e., intercept = 0, slope = 1).

**Fig 6:**
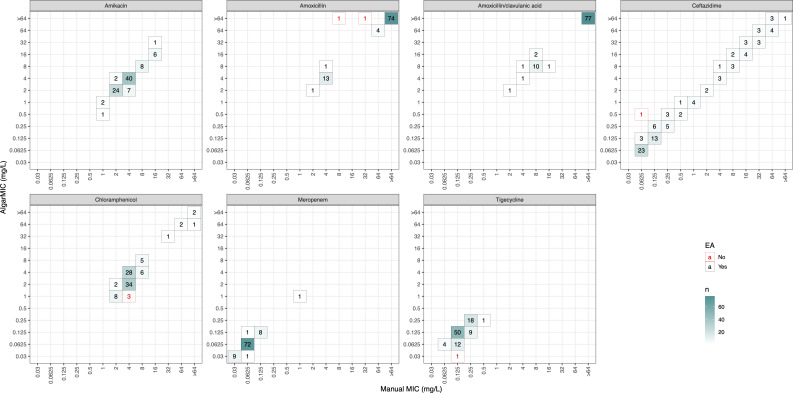
Heatmap of MIC results for AIgarMIC against manual annotation on *E. coli* strains, EA, essential agreement.

The end-to-end performance of the model was assessed by comparing the algorithm output to the data set of manually annotated MIC results. After excluding strains and antibiotics that failed quality control (i.e., the absence of growth in control plate without antibiotics, or control strain MIC outside of recommended range), 651 MIC results were analyzed (Fig. 5 ). Of these, 644/651 (98.9%) had essential agreement between the AI-generated MIC and manually validated controls (above 90% is deemed acceptable by ISO 20776-2:2021) (19). A complete breakdown of each individual agent’s MIC results is available in Fig. 6. The AI method had a bias of −7.8% which is within the acceptable range of ±30% specified by ISO 20776-2:2021 (19). Error rates were low, with 1 (0.2%) minor error, 3 (0.5%) major errors, and 1 (0.2%) very major error. These results were reflected across all the evaluated antimicrobials, except for chloramphenicol, which had a bias of −55.4%.

## DISCUSSION

Accurate classification of phenotypic susceptibility to antimicrobial agents is a critical tool for the appropriate clinical therapeutic use of antimicrobials at both an individual patient and population level. Construction of MIC distributions provides information that guides surveillance programs and, therefore, informs local, regional, national, and international regimen planning (21). The deployment of conventional MIC testing methods at scale is hindered by their low throughput, intensive clinical scientist input, and high consumables usage. Although there are various automated and semi-automated solutions available, many carry prohibitively high up-front and/or maintenance costs (22–27).

By harnessing modern ML and AI quantitative techniques, AIgarMIC augments and semi-automates the agar dilution MIC calculation process. Our evaluation shows that even when trained on a modest data set of 1,055 colony images, AIgarMIC performs well when compared to manual annotation. This approach offers immediate advantages in time saved in result interpretation and reporting, and reduction of human error.

The two-step model approach provided additional computational efficiency by tailoring model complexity to the prior likely complexity of the classification problem. A simpler model can be used to determine whether any growth is present, reserving more complex models to the more subtle problem of differentiating between uninhibited growth and antibiotic-impaired growth, making more efficient use of computational resources.

In agar dilution MIC, identification of the antibiotic concentration that leads to bacterial growth inhibition is often subjective—the visual appearance of the colony is affected by the drug-pathogen combination and the experimental conditions used to estimate MIC (10). Incorrectly labeling plates with inhibited growth as uninhibited growth may lead to an incorrect MIC (one or more dilutions higher than the correct test MIC). Some of this subjectivity also affects the image training data set annotation, as evidenced by the sub-100% classification accuracy of the final trained models (Fig. 4). Although difficult to quantify, this subjectivity likely contributes to intra- and inter-laboratory variability of MIC measurement (28, 29). AIgarMIC standardizes this subjectivity since it is trained on a fixed data set and, therefore, can be considered a single fixed interpretative operator. A further advantage of the image recognition-based method is that images can be stored in perpetuity to produce a long-term record of the results.

Agar dilution protocols instruct the reader to disregard isolated colonies and faint film (or haze) growth (10, 12). Our approach was to label colonies with such features within a separate class (poor/inhibited growth) and count such colonies as “no growth” for the purpose of MIC calculation. Alternative approaches are possible, such as only having two classes (growth and no growth), with poor/inhibited growth colonies assigned to the no growth class. Although this could lead to marginal computational efficiency gains, we expect that colonies around the MIC would be subject to mislabeling and incorrect MICs. Nevertheless, the assessment of the performance of simpler machine-learning models should be explored in future research. A secondary advantage of having a specific poor/inhibited growth class is the flexibility offered for future models in dealing with bug-drug combinations that tend to produce variable morphology around the MIC (such as chloramphenicol in this study). Models could be calibrated toward classifying uncertain images as good growth or poor growth, to reduce bias.

AIgarMIC uses agar dilution as its underlying wet laboratory procedure—a standard method with a long history and referenced by international susceptibility testing organizations (2, 12). Further end-to-end validation against an alternative reference method (broth microdilution) is desirable prior to routine use in clinical settings. The validation data produced by this study is for a limited number of drug-pathogen combinations and was performed in one laboratory—further validation data from multiple laboratories is, therefore, required. Since only *E. coli* strains were used in the validation of AIgarMIC, future work should focus on the performance of the model on organisms not evaluated within this study, such as gram-positive, anaerobic, and organisms with fastidious growth requirements.

AIgarMIC has practical limitations inherent to agar dilution; for example, only one antimicrobial concentration (or specific combination such as beta-lactamase & beta-lactamase inhibitor) can be added to each agar plate; additional antibiotics require a full set of plates for the required concentration range. Therefore, the method is better suited to testing many strains against fewer antimicrobials. Chloramphenicol MICs were biased toward under-reporting MIC. On revisiting images of chloramphenicol MICs, a faint film of growth was common around the MIC (Fig. 3 – second row, fourth image), which is challenging to differentiate from good growth and likely explains the bias. Chloramphenicol also had the highest number of essential agreement failures (3/92, 3.3%). This warrants further research and evaluation, such as more training data or training of a separate neural network for this agent. Furthermore, drug-pathogen combinations with strict growth medium requirements (e.g., cefiderocol) are unsuitable for agar dilution (30).

The utility of AIgarMIC is primarily in the analytical and post-analytical stages of MIC testing; the required baseline laboratory capacity in the wet laboratory process remains substantial. The advantages provided by AIgarMIC, however, could bring MIC testing closer to the organism—for example, agar could be prepared in a central location and then shipped to the testing laboratory to reduce the need for inter-laboratory transport of pathogenic bacteria.

AIgarMIC is suited to a variety of applications—especially in laboratories where access to automated MIC testing is limited. No specialist imaging or computing equipment is required—the model is trained and validated on a consumer-grade photographic camera and laptop computer. An advantage of a neural network approach is that the model can work with (and, indeed, was trained on) images with imperfect lighting conditions and imaging angles. Depending on the chosen application, the user can be prompted to confirm and override key images with poor model prediction, therefore increasing the users’ trust in the results. Since images are used for MIC calculation, the laboratory process can be separated from annotation and reporting, allowing agar dilution to be carried out in laboratories without the required on-site expertise for MIC reporting.

AIgarMIC demonstrates the potential impact of novel ML quantitative techniques becoming more accessible. Older, but more established, laboratory techniques can be re-imagined to explore applications beyond their current scope.

## Data Availability

AIgarMIC and associated source code can be accessed on GitHub at https://github.com/agerada/AIgarMIC.

## SUPPLEMENTAL MATERIAL

Supplemental material spectrum.04209-23-s0001.docx Data S1, Table S2, Figures S3 and S4, and Data S5.

ASM does not own the copyrights to Supplemental Material that may be linked to, or accessed through, an article. The authors have granted ASM a non-exclusive, world-wide license to publish the Supplemental Material files. Please contact the corresponding author directly for reuse.
