# Supplementary material for: Determination of minimum inhibitory concentrations using machine-learning-assisted agar dilution
Source: Microbiol Spectr. 2024 Mar 22;12(5):e04209-23. doi: 10.1128/spectrum.04209-23 (PMC11064640; doi:10.1128/spectrum.04209-23)
Supplement: Supplemental material — Data S1, Table S2, Figures S3 and S4, and Data S5. [file spectrum.04209-23-s0001.docx]

Supplementary material

Manuscript title Determination of minimum inhibitory concentrations using machine learning-assisted agar dilution

Table of Contents

[Supplementary Data S1 – validation antimicrobials concentrations and QC data 2](#_Toc153968828)

[Supplementary Table S2 – neural network test/train image file numbers 3](#_Toc153968829)

[Supplementary Figure S3– neural network architecture 4](#_Toc153968830)

[Supplementary Figure S4 – neural network sigmoid output layer plot 5](#_Toc153968831)

[Supplementary Data S5 – Equipment specifications 7](#_Toc153968832)

# Supplementary Data S1 – validation antimicrobials concentrations and QC data

| **Antimicrobial** | **Concentrations tested (mg/L)** | **QC organism** | **QC MIC range (mg/L)^[[1]](#footnote-2)^** | **QC MIC result (mg/L)^[[2]](#footnote-3)^** |
| --- | --- | --- | --- | --- |
| Amikacin | 64, 32, 16, 8, 4, 2, 1, 0.5, 0.25, 0.125, 0.06, 0.03, 0 (control) | ATCC *E. coli* 25922 | 0.5–4 | 2.0 |
| Amoxicillin | 64, 32, 16, 8, 4, 2, 1, 0.5, 0.25, 0.125, 0.06, 0.03, 0.015, 0 (control) | ATCC *E. coli* 25922 | 2–8 | 4.0 |
| Amoxicillin/clavulanic acid | 64, 32, 16, 8, 4, 2, 1, 0.5, 0.25, 0.125, 0.06, 0.03, 0.015, 0 (control) | ATCC *E. coli* 25922 | 2–8 | 8.0 |
| Ceftazidime | 64, 32, 16, 8, 4, 2, 1, 0.5, 0.25, 0.125, 0.06, 0.03, 0.015625, 0 (control) | ATCC *E. coli* 25922 | 0.06–0.5 | 0.06 |
| Chloramphenicol | 64, 32, 16, 8, 4, 2, 1, 0.5, 0.25, 0.125, 0.06, 0.03, 0 (control) | ATCC *E. coli* 25922 | 2–8 | 4.0 |
| Meropenem | 64, 32, 16, 8, 4, 2, 1, 0.5, 0.25, 0.125, 0.06, 0.03, 0.015, 0.008, 0.004, 0 (control) | ATCC *E. coli* 25922 | 0.008–0.06 | 0.06 |
| Tigecycline | 64, 32, 16, 8, 4, 2, 1, 0.5, 0.25, 0.125, 0.06, 0.03, 0.015, 0.008, 0 (control) | ATCC *E. coli* 25922 | 0.03–0.25 | 0.125 |

# Supplementary Table S2 – neural network test/train image file numbers

Table S2: Number of image files used for neural network training and testing

| Class | Training dataset (n) | Testing dataset (n) | Totals (training + testing) |
| --- | --- | --- | --- |
| No growth | 142 | 35 | 177 |
| Poor growth | 335 | 84 | 419 |
| Good growth | 578 | 145 | 723 |
| Totals (all classes) | 1055 | 264 | 1319 |

# Supplementary Figure S3– neural network architecture

Figure S1 - Neural network architecture for both binary classification models - any growth versus no growth (left), good growth versus inhibited growth (right)

# Supplementary Figure S4 – neural network sigmoid output layer plot

Figure 2 - Neural network final sigmoid layer output of validation dataset images. The binary classification neural network outputs a value between 0 and 1, which is classified using a threshold of 0.5. Incorrect classifications cluster around the 0.5 threshold.

# Supplementary Data S5 – Equipment specifications

Images were photographed using:

Canon EOS 4000D camera

Tripod

Imaging box

Back lighting

Front lighting (optional)


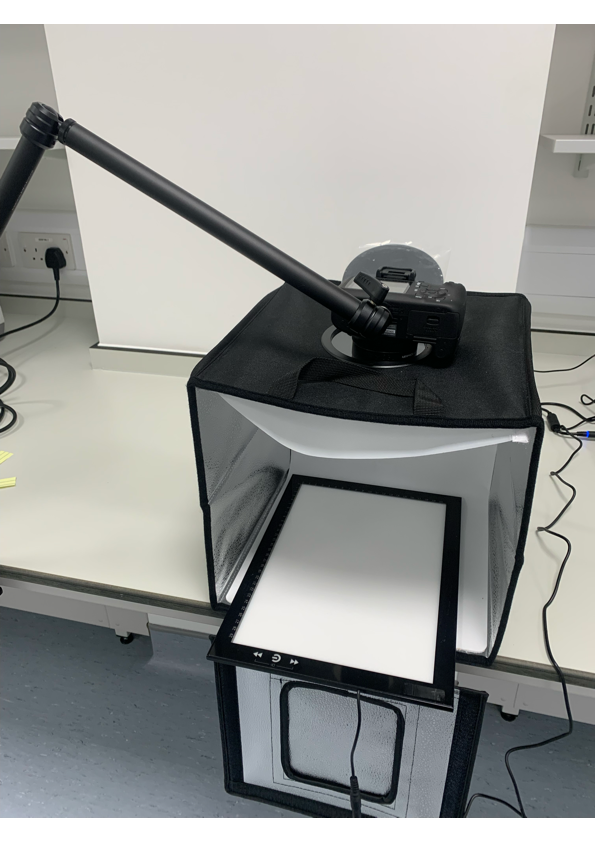


Figure 3 – Imaging equipment setup

Computer used for model development and testing:

Apple M1 MacBook Pro 8Gb

1. The European Committee on Antimicrobial Susceptibility Testing. Routine and extended internal quality control for MIC determination and disk diffusion as recommended by EUCAST.
   Version 13.2, 2023. http://www.eucast.org [↑](#footnote-ref-2)
2. One QC strain was required per antimicrobial tested, inoculated on the last spot of the 96-spot agar plate [↑](#footnote-ref-3)
